# A VVWBO-BVO-based GM (1,1) and its parameter optimization by GRA-IGSA integration algorithm for annual power load forecasting

**DOI:** 10.1371/journal.pone.0196816

**Published:** 2018-05-16

**Authors:** Lianhui Li, Hongguang Wang

**Affiliations:** 1 College of Mechatronic Engineering, North Minzu University, Yinchuan, China; 2 The 713th Research Institute of China Shipbuilding Industry Corporation, Zhengzhou, China; Southwest University, CHINA

## Abstract

Annual power load forecasting is not only the premise of formulating reasonable macro power planning, but also an important guarantee for the safety and economic operation of power system. In view of the characteristics of annual power load forecasting, the grey model of GM (1,1) are widely applied. Introducing buffer operator into GM (1,1) to pre-process the historical annual power load data is an approach to improve the forecasting accuracy. To solve the problem of nonadjustable action intensity of traditional weakening buffer operator, variable-weight weakening buffer operator (VWWBO) and background value optimization (BVO) are used to dynamically pre-process the historical annual power load data and a VWWBO-BVO-based GM (1,1) is proposed. To find the optimal value of variable-weight buffer coefficient and background value weight generating coefficient of the proposed model, grey relational analysis (GRA) and improved gravitational search algorithm (IGSA) are integrated and a GRA-IGSA integration algorithm is constructed aiming to maximize the grey relativity between simulating value sequence and actual value sequence. By the adjustable action intensity of buffer operator, the proposed model optimized by GRA-IGSA integration algorithm can obtain a better forecasting accuracy which is demonstrated by the case studies and can provide an optimized solution for annual power load forecasting.

## Introduction

### Motivation

Accurate power load forecasting is the key to realize the sustainable development of the power industry and ensure the reliable and safe operation of the power grid. In the past few years, many scholars have given a lot of attention to short-term power load forecasting based on different factors and have reached a higher level. Annual power load forecasting can provide reliable guidance for power grid operation and power construction planning [1–4]. Affected by economic, climate and other factors, an annual load curve often shows a non-linear characteristic and has a certain degree of mutation. As a result, the difficulty of annual power load forecasting is obviously larger than short-term power load prediction.

### Literature review

In recent years, different kinds of power load forecasting technology have been developed to improve the forecasting accuracy. The traditional forecasting methods, such as time series analysis [5,6], regression model [7] and auto-regressive moving average model [8], have good forecasting accuracy, but the nonlinear simulating ability is poor. With the development of intelligent technology, many new intelligent methods have been used for annual power load forecasting. Bozkurt et al. [9] introduced two different models for current Turkish Market using Seasonal Autoregressive Integrated Moving Average (SARIMA) and Artificial Neural Network (ANN) and presented an Artificial neural network and SARIMA based models for power load forecasting. Lang et al. [10] proposed a new forecasting model based on the improved neural networks with random weights (INNRW). Fu et al. [11] combined the fruit fly optimization algorithm with the grey neural network and finally improved the prediction accuracy, but this method was easy to fall into local optimal or premature convergence.

On the whole, annual power load of an area is growing year by year. However, due to the impact of various external factors, such as economic policy and weather condition, there are a variety of random fluctuations in the growth process of annual power load. According to the grey theory proposed by Deng [12], annual power load forecasting can be regarded as a typical grey system. Therefore, the GM (1,1) model and its optimization model have been widely applied in annual power load prediction and other similar fields [13–20]. Representative researches are as follows. Zhao et al. [13] put forward a new hybrid electricity consumption forecasting method based on GM (1,1) optimized by moth-flame optimization (MFO) algorithm with rolling mechanism. Karadede et al. [18] showed a general natural gas demand forecasting model of the breeder hybrid algorithm based nonlinear regression and applied it to Turkey natural gas demand forecasting to show its superiority and applicability. Boroojeni et al. [19] presented a generalized technique to model historical load data in the form of time-series with different cycles of seasonality for electric power demand forecasting. In day-ahead natural gas demand predictions, Panapakidis et al. [20] tested the robustness of a novel hybrid computational intelligence model, which combined the Wavelet Transform (WT), Genetic Algorithm (GA), Adaptive Neuro-Fuzzy Inference System (ANFIS) and Feed-Forward Neural Network (FFNN).

If the forecasting model is directly constructed based on original annual power load data, it may lead to a larger forecasting error, or the simulating effect is good but the forecasting effect is poor. For this reason, Liu [21] proposed the concept of buffer operator and established the GM (1,1) model after using the buffer operator to pre-process the historical annual power load data. So the smoothness of the modeling sequence is effectively improved, the randomness of the historical annual power load data is weakened and the overall trend of load development is strengthened, and the accuracy of GM (1,1) model is improved. However, the buffer operator still has problems in the practical load forecasting application. The traditional buffer operator is not adjustable in intensity. According to the qualitative analysis and the subjective judgment, users can only select a fixed buffer operator which is less adaptable, so the buffer effect is sometimes too weak and sometimes too strong. If the buffer effect is too weak, the pre-process effect will be not obvious and the forecasting accuracy of the model will be limited. If the buffer effect is too strong, the inherent variation of the historical annual power load sequence will be changed. Although high accuracy simulating of the pre-process load sequence can be achieved, the forecasting result will actually deviate from the initial annual power load forecasting problem.

### Our contribution

Aiming at the above practical application problems of traditional GM (1,1) forecasting model in annual power load forecasting, variable-weight weakening buffer operator (VWWBO) and background value optimization (BVO) are introduced into GM (1,1) and a VWWBO-BVO-based GM (1,1) is proposed. Next, grey relational analysis (GRA) and improved gravitational search algorithm (IGSA) are integrated for the parameter optimization of proposed model. By the adjustable action intensity of weakening buffer operator, the dynamic pre-process of historical annual power load data is realized and the forecasting accuracy is improved. Meanwhile, the inherent trend and change rule of the original power load sequence are maintained to the maximum extent in the forecasting and the stability of simulating and forecasting are improved.

### Organization of paper

The rest of this paper is organized as follows. Details of the methods, which we used to create our model, are given in Methods. Case studies and detailed discussion on experimental results are presented in Experimental results. Finally, we conclude the paper and provide guidelines for future work in Conclusions.

## Methods

### Basic knowledge of the traditional GM (1,1) forecasting model

Based on the known or uncertain information of past and present, grey theory [12, 21] is aiming to build a grey model from past to future, so as to determine the trend of future development of the system. Any random process is considered to be a grey amount that changes within a certain range in grey theory. By accumulating the irregular original power data sequence, a new sequence of randomness weakening and regularity strengthening will be generated. The grey model of GM (1,1) is a differential equation model based on the generated sequence. The specific implementation steps of GM (1,1) are given below.

**Step 1**. The original power data sequence is given as follows:
  $$x^{\left(0\right)}=(x^{\left(0\right)}(1),x^{\left(0\right)}(2),\ldots ,x^{\left(0\right)}(n))$$ (1)
  where *x*^(0)^(*t*) ≥ 0, *t* = 1,2,…, *n*.
  1-accumulating generation operator (1-AGO) is used to generate the one-order accumulative sequence, that is:
  $$x^{\left(1\right)}=(x^{\left(1\right)}(1),x^{\left(1\right)}(2),\ldots ,x^{\left(1\right)}(n))$$ (2)
  where $x^{\left(1\right)}(t)=\sum_{k=1}^{t}x^{\left(0\right)}(k),t=1,2,\ldots ,n$. *x*^(1)^ is named as the 1-AGO sequence of *x*^(0)^.
**Step 2**. For *x*^(1)^(*t*), its one-order differential equation is constructed as follows:
  $$\frac{\text{d}x^{\left(1\right)}}{\text{d}t}+ax^{\left(1\right)}=u$$ (3)
  where *a* and *u* are undetermined coefficients and called the development coefficient and the grey amount of action, respectively. The effective interval of *a* is (-2, 2).
  The matrix consisting of *a* and *u* is defined as the grey parameter as follows:
  $$\hat{A}={\left(a,u\right)}^{\text{T}}$$ (4)
  *x*^(1)^(*t*) can be calculated just by calculating the value of *a* and *u*, and then the forecasted value of *x*^(0)^ can be calculated.
  For *x*^(1)^, the mean generation vector *B* and the constant term vector *Y* are constructed as follows:
  $$B={\left(\frac{1}{2}(x^{\left(1\right)}(1)+x^{\left(1\right)}(2)),\frac{1}{2}(x^{\left(1\right)}(2)+x^{\left(1\right)}(3)),\ldots ,\frac{1}{2}(x^{\left(1\right)}(n-1)+x^{\left(1\right)}(n))\right)}^{\text{T}}$$ (5)
  $$Y={\left(x^{\left(0\right)}(2),x^{\left(0\right)}(3),\ldots ,x^{\left(0\right)}(n)\right)}^{\text{T}}$$ (6)
  The least square method is used to solve the grey parameter $\hat{A}$, that is:
  $$\hat{A}\approx {\left(\hat{a},\hat{u}\right)}^{\text{T}}={\left(B^{\text{T}}B\right)}^{-1}B^{\text{T}}Y$$ (7)
  where $\hat{a}$ and $\hat{u}$ are the approximate value of *a* and *u*, respectively.
**Step 3**. The obtained grey parameter $\hat{A}$ is substituted into Eq (3) and Eq (3) is solved as follows:
  $${\hat{x}}^{\left(1\right)}(t+1)=(x^{\left(0\right)}(1)-\frac{\hat{u}}{\hat{a}})e^{-\hat{a}t}+\frac{\hat{u}}{\hat{a}}$$ (8)
  Here, because $\hat{A}$ is an approximate value obtained by the least square method, ${\hat{x}}^{\left(1\right)}(t+1)$ is also an approximate expression.
  The original power data sequence *x*^(0)^ can be restored by ${\hat{x}}^{\left(1\right)}(t+1)$ and ${\hat{x}}^{\left(1\right)}(t)$, and the approximate data sequence ${\hat{x}}^{\left(0\right)}(t+1)$ can be obtained as follows:
  $${\hat{x}}^{\left(0\right)}(t+1)={\hat{x}}^{\left(1\right)}(t+1)-{\hat{x}}^{\left(1\right)}(t)$$ (9)
**Step 4**. The accuracy of the established GM (1,1) model is checked as follows.
  Between ${\hat{x}}^{\left(0\right)}(t)$ and *x*^(0)^(*t*), the residual is $\varepsilon^{\left(0\right)}(t)=x^{\left(0\right)}(t)-{\hat{x}}^{\left(0\right)}(t)$ and the relative error which is also called Percentage Error (PE) is Δ^(0)^(*t*) = *ε*^(0)^(*t*)/*x*^(0)^(*t*). So Mean Absolute Percentage Error (MAPE) is $\theta =\frac{1}{n}\sum_{t=1}^{n}\left|\Delta^{\left(0\right)}(t)\right|$.
**Step 5**. The established GM (1,1) model is used for forecasting as follows:
  $${\hat{x}}^{\left(0\right)}=(\underset{\text{Simulation}\;\text{of}\;\text{original}\;\text{data}\;\text{sequence}}{\underset{︸}{{\hat{x}}^{\left(0\right)}(1),{\hat{x}}^{\left(0\right)}(2),\ldots ,{\hat{x}}^{\left(0\right)}(n)}},\underset{\text{Forecast}\;\text{of}\;\text{future}\;\text{data}\;\text{sequence}}{\underset{︸}{{\hat{x}}^{\left(0\right)}(n+1),{\hat{x}}^{\left(0\right)}(n+2),\ldots ,{\hat{x}}^{\left(0\right)}(n+m)}})$$ (10)

### The proposal of VWWBO-BVO-based GM (1,1)

Generally, the grey model of GM (1,1) has many advantages, such as less load data, no need to consider distribution rules, easy operation and test. However, there are some limitations in its application of annual power load forecasting. In the original power data sequence of load forecasting, the former part is growing faster, but the latter part is growing slowly. It’s not a strict regular sequence. The original power data sequence cannot correctly reflect the real change rule because of the impact disturbance.

By the effect of the weakening buffer operator on the original power data sequence, the effect of the exception value is weakened, and the change trend of the original data is strengthened. In addition, the weakening buffer operator satisfies the principle of full utilization of information and the principle of "new information priority", that is, the new data sequence is generated by the buffer operator on the basis of the information in the existing data sequence, and the latest information remains unchanged under the action of the buffer operator.

Therefore, using the weakening buffer operator on the original power data sequence can weaken the impact of interference data and strengthen the function of the latest information. As a result, the problem of inconsistent quantitative forecasting result and qualitative analysis conclusion can be effectively solved [21].

The action intensity of the traditional buffer operator is nonadjustable and the fixed buffer operator is less adaptable. This will lead to an uncertain buffer effect, so we introduce VWWBO into the traditional GM (1,1) forecasting model for the dynamic pre-process of historical annual power data.

The original power load data sequence is *x* = (*x*(1), *x*(2),…, *x*(*q*)) and the variable-weight weakening buffer operator is *D*. After the pre-processing by *D*, *x* is converted to *y* as follows:

y=xD=(y(1),y(2),…,y(q)) (11)

where *y*(*k*) = *x*(*k*)*d* = *λx*(*q*) + (1−*λ*)*x*(*k*), 0 ≤ *λ* ≤ 1, *x*(*k*) > 0, *k* = 1,2,…, *q*.

Whenever *x* is a monotone increasing sequence, a monotone reducing sequence or an oscillating sequence, *D* is always a weakening buffer operator [15]. Here, *λ* is the variable-weight buffer coefficient.

The average change rate from *x*(*k*) to *x*(*q*) in *x* is represented as *r*(*k*):

$$r(k)=\frac{x(q)-x(k)}{q-k+1}$$ (12)

The adjustment degree of *D* at *k* is represented as *δ*(*k*):

$$\delta (k)=\left|\frac{r(k)-r(k)d}{r(k)}\right|$$ (13)

The adjustment degree of *D* at each point is a constant which equals to the variable-weight weakening buffer coefficient *λ* (i.e., *δ*(*k*) = *λ*) [15]. The adjustment degree *δ*(*k*) reflects the buffer operator’s effect on the original power load data sequence. By dynamically adjusting the variable-weight buffer coefficient *λ*, it can realize the fine-tuning of the buffer operator’s effect and effectively solve the problem of traditional fixed buffer operator. As a result, the flexibility, controllability and adaptability of the buffer operator in the pre-processing of power load data sequence have been enhanced.

In order to lighten the fluctuation of original power load data sequence, strengthen the trend of modeling sequence and improve the forecasting accuracy, VWWBO and BVO [17] are used to improve the traditional GM (1,1) forecasting model. So, the VWWBO-BVO-based GM (1,1) is built as follows.

**Step 1**. The historical annual power load data sequence is *x*^(0)^ = (*x*^(0)^(1), *x*^(0)^(2),…,*x*^(0)^(*q*)). The variable-weight weakening buffer operator *D* is used to pre-process *x*^(0)^. After the pre-processing, *x*^(0)^ is converted to *y*^(0)^ = (*y*^(0)^(1),*y*^(0)^(2),…,*y*^(0)^(*q*)) where *y*^(0)^(*t*) = *λx*^(0)^(*q*) + (1−*λ*),*x*^(0)^(*t*) and *t* = 1,2,…,*q*.
**Step 2**. For *y*^(0)^, 1-AGO is used to generate the one-order accumulative sequence, that is *y*^(1)^ = (*y*^(1)^(1),*y*^(1)^(2),…,*y*^(1)^(*q*)) where $y^{\left(1\right)}(t)=\sum_{k=1}^{t}y^{\left(0\right)}(k)$ and *t* = 1,2,…,*q*. Next, the variable-weight background value *z*^(1)^ is constructed as *z*^(1)^ = (*z*^(1)^(2),*z*^(1)^(3),…,*z*^(1)^(*q*)) where *z*^(1)^(*t*) = *ηy*^(1)^(*t*−1) + (1−*η*),*y*^(1)^(*t*) and *t* = 2,3,…,*q*, here *η* is the background-value weight generating coefficient, 0 ≤ *η* ≤ 1.
**Step 3**. The GM (1,1) model is built as *y*^(0)^(*t*) + *az*^(1)^(*t*) = *u* where the parameters are *a* and *u*. The grey parameter $\hat{A}={\left(a,u\right)}^{\text{T}}$ can be solved by the least square method, that is $\hat{A}={\left[\hat{a},\hat{u}\right]}^{\text{T}}={\left(B^{\text{T}}B\right)}^{-1}B^{\text{T}}Y$ where:
  $$\left\{ \begin{array}{l} B=\left[\begin{array}{cc} -z^{\left(1\right)}(2) & 1\\ -z^{\left(1\right)}(3) & 1\\ \vdots & \vdots \\ -z^{\left(1\right)}(q) & 1 \end{array}\right]\\ Y=\left[\begin{array}{c} y^{\left(0\right)}(2)\\ y^{\left(0\right)}(3)\\ \vdots \\ y^{\left(0\right)}(q) \end{array}\right] \end{array}\right.$$ (14)
**Step 4**. The solution of the model is obtained as ${\hat{y}}^{\left(1\right)}=({\hat{y}}^{\left(1\right)}(1),{\hat{y}}^{\left(1\right)}(2),\ldots ,{\hat{y}}^{\left(1\right)}(q))$ where ${\hat{y}}^{\left(1\right)}(t)=(y^{\left(0\right)}(1)-\frac{\hat{u}}{\hat{a}})e^{-\hat{a}(t-1)}+\frac{\hat{u}}{\hat{a}}$ and *t* = 1,2,…,*q*.
**Step 5**. The forecasting value is obtained as ${\hat{y}}^{\left(0\right)}=({\hat{y}}^{\left(0\right)}(1),{\hat{y}}^{\left(0\right)}(2),\ldots ,{\hat{y}}^{\left(0\right)}(q))$ where ${\hat{y}}^{\left(0\right)}(t)={\hat{y}}^{\left(1\right)}(t)-{\hat{y}}^{\left(1\right)}(t-1)$ and *t* = 2,3,…,*q*.

Therefore, the above VWWBO-BVO-based GM (1,1) is a development of the traditional GM (1,1) model. It will degenerate into the traditional GM (1,1) model if and only if *λ* = *η* = 0.5.

### GRA-IGSA integration algorithm for model parameter optimization

#### GSA algorithm

The key factors that affect the forecasting accuracy of the above VWWBO-BVO-based GM (1,1) are the variable-weight weakening buffer coefficient *λ* and the background-value weight generating coefficient *η*. A GRA-IGSA integration algorithm is proposed to find the optimal value of *λ* and *η*.

**Fig 1:**
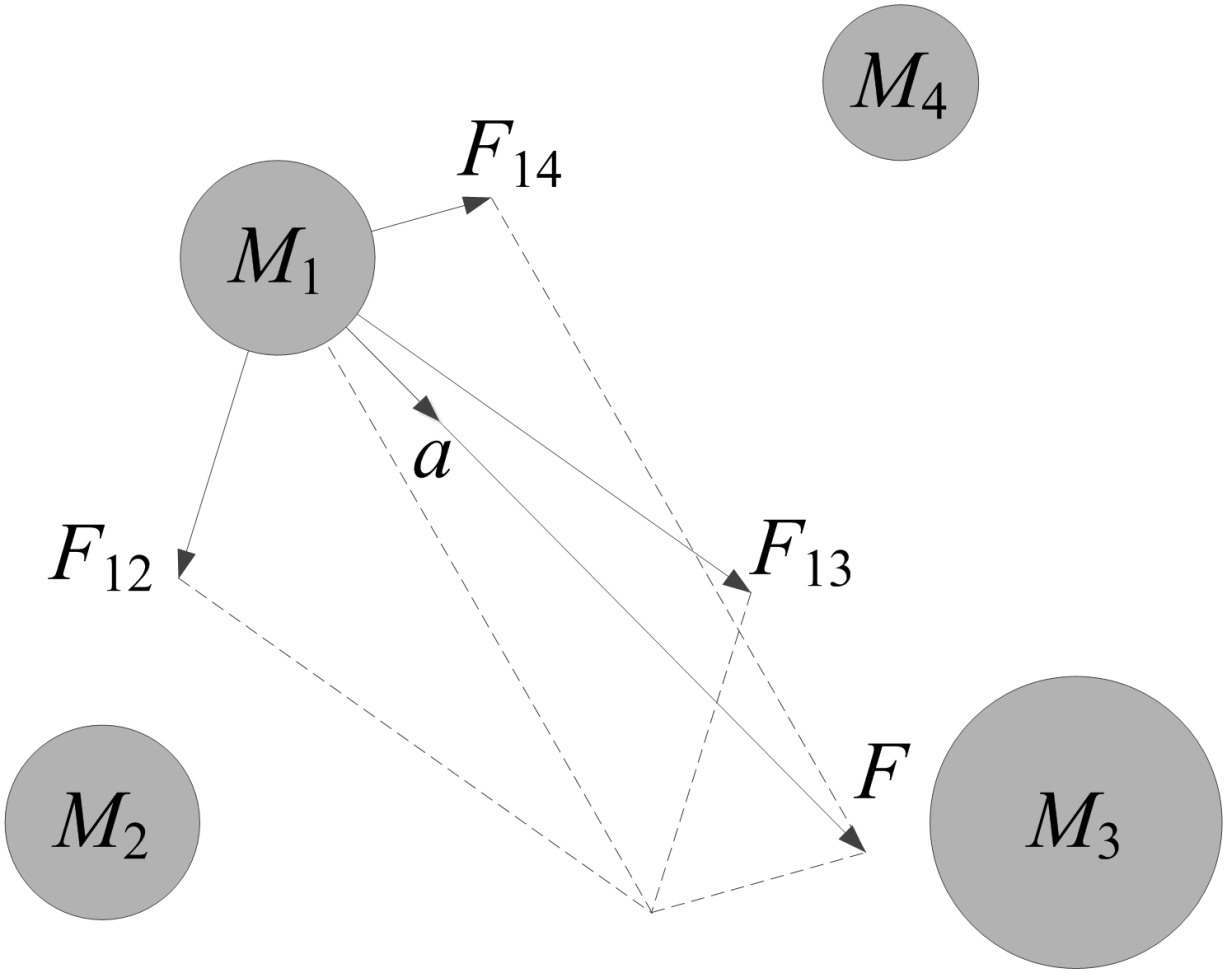
Gravitational motion mechanism.

In 2009, Rashedi et al. [22] proposed the gravitation search algorithm (GSA). GSA is a new meta-heuristic algorithm and has been used to solve nonlinear benchmark function problem [23] and nonlinear filtering modeling problem [24]. In GSA [25–27], each object has the position and velocity which represent the solution. The mass of an object is determined by its position and fitness value. An object with a better fitness value has a greater mass. From the gravitational motion mechanism shown in Fig 1, an object with a greater mass will generate a greater gravitation. This will lead to the motion of small mass objects toward large mass objects, so the information interaction among objects and population evolution is realized. In Fig 1, *F*, *M* and *a* are the symbols of force, mass and acceleration respectively.

For a system consisting of *N* objects, in gravitational search, the position *P*_*i*_ and the velocity *V*_*i*_ of the object *i* (*i* = 1,2,…,*N*) are defined as:

$$P_{i}=(p_{i}^{1},p_{i}^{2},\ldots ,p_{i}^{L}),V_{i}=(v_{i}^{1},v_{i}^{2},\ldots ,v_{i}^{L})$$ (15)

where *L* is the dimension number of search space, $p_{i}^{l}$ and $v_{i}^{l}$ are the position and velocity of the object *i* in the dimension *l* and *l* = 1,2,…,*L*.

On a specific time *T*, the hypotheses are as follows:

- The mass of the object *i* and the object *j* are *M*_*i*_(*T*) and *M*_*j*_(*T*), respectively.
- The gravitational constant is *G*(*T*) and the Euclidean distance between the object *i* and the object *j* is *R*_*ij*_(*T*).
- The positions of the object *i* and the object *j* are *P*_*i*_(*T*) and *P*_*j*_(*T*), respectively.
- The positions of the object *i* and the object *j* in dimension *l* are $p_{i}^{l}(T)$ and $p_{j}^{l}(T)$, respectively.

Therefore, the gravitation acting on the object *i* by the object *j* on the time *T* can be defined as:

$$F_{ij}(T)=(F_{ij}^{1}(T),F_{ij}^{2}(T),\ldots ,F_{ij}^{L}(T))$$ (16)

where the component of *F*_*ij*_(*T*) in the dimension *l* (*l* = 1,2,…,*L*) is $F_{ij}^{l}(T)=G(T)\frac{M_{i}(T)M_{j}(T)}{R_{ij}(T)+\xi }(p_{j}^{l}(T)-p_{i}^{l}(T))$. Here, *ξ* is a small constant and *R*_*ij*_(*T*) = ∥*P*_*i*_(*T*),*P*_*j*_(*T*)∥_2_.

To increase the random characteristics of the algorithm, the resultant of forces acting on the object *i* in the dimension *l* is defined as follows:

$$F_{i}^{l}(T)=\sum_{j=1,j\neq i}^{N}rand_{j}F_{ij}^{l}(T)$$ (17)

where *rand*_*j*_ is a random variable in the interval [0, 1].

The acceleration of the object *i* in the dimension *l* on the time *T* is as follows:

$$a_{i}^{l}(T)=\frac{F_{i}^{l}(T)}{M_{i}(T)}$$ (18)

Therefore, the position and the velocity of the object *i* in the dimension *l* on the next time *T*+1 are as follows:

$$p_{i}^{l}(T+1)=p_{i}^{l}(T)+v_{i}^{l}(T+1),v_{i}^{l}(T+1)=rand_{i}\cdot v_{i}^{l}(T)+a_{i}^{l}(T)$$ (19)

where *rand*_*i*_ is a random variable in the interval [0, 1].

The mass of each object is computed first by the fitness function values shown in Eq (20), and then normalized by Eq (21):

$$m_{i}(T)=\frac{f_{P_{i}}(T)-f_{worst}(T)}{f_{best}(T)-f_{worst}(T)}$$ (20)

$$M_{i}(T)=\frac{m_{i}(T)}{\sum_{j=1}^{N}m_{j}(T)}$$ (21)

where $f_{P_{i}}(T)$ is the fitness value of the object *i* on the time *T*, *f*_*best*_(*T*) and *f*_*worst*_(*T*) are the best and worst fitness value on the time *T*, respectively.

Gravitational constant, which plays a very important role in GSA, is defined as a function of the time *T*:

$$G(T)=G_{0}\cdot \text{exp}(-s\cdot \frac{T}{I})$$ (22)

where *G*_0_ is the initial value, *s* is a constant, *I* is the number of iterations. From Eq (22), we can see that the value of gravitational constant decreases as the number of iterations increases. Thus the search stride is reduced and the search accuracy is improved.

#### IGSA algorithm based on local search

**Fig 2:**
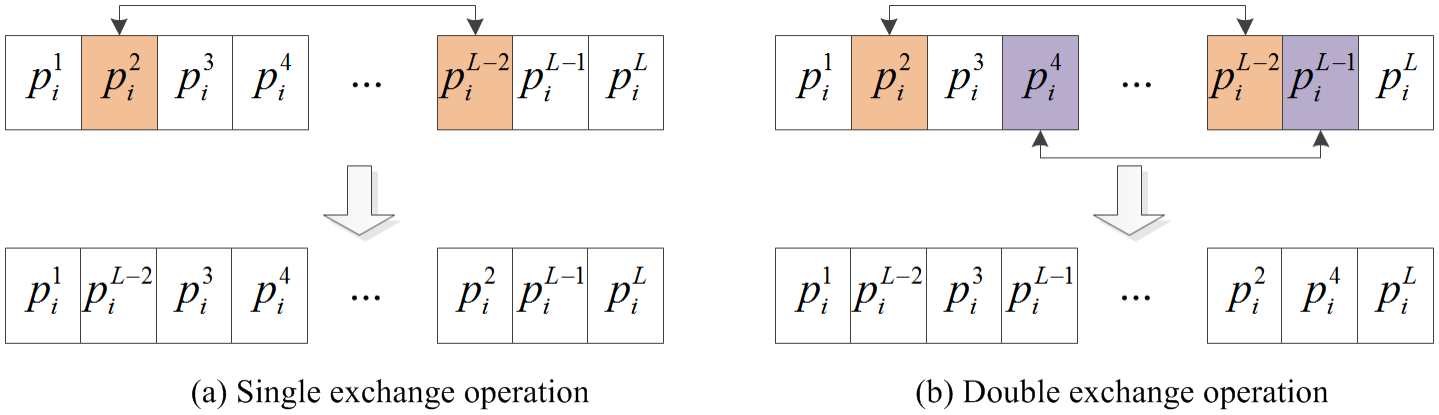
Exchange operation.

GSA has a fast convergence rate, but it is easy to fall into local optimum. In order to develop the local search capability, a local search algorithm is introduced to increase the chance of better solution and improve GSA. In the local search algorithm, single exchange operator and double exchange operator are used to generate a new neighborhood structure and then new solution is obtained. The exchange operation is shown in Fig 2. In single exchange operation shown in Fig 2(a), the positions of object *i* in two random dimensions are chosen and exchanged. In double exchange operation shown in Fig 2(b), single exchange operator is done twice.

The local search algorithm that incorporates the exchange operators follows below steps:

**Step 1**. Begin with *i* = 1, $P_{i}=(p_{i}^{1},p_{i}^{2},\ldots ,p_{i}^{L})$ represents the solution of the object *i*.
**Step 2**. If the random number *rand* < 0.5, do the single exchange operation and obtain a new solution $P_{i}^{*}$, turn Step 4. Otherwise, turn Step 3.
**Step 3**. Do the double exchange operation and obtain a new solution $P_{i}^{*}$.
**Step 4**. Calculate the fitness function $f_{P_{i}^{*}}$.
**Step 5**. If $f_{P_{i}^{*}}\leq f_{P_{i}}$, accept $P_{i}^{*}$. Replace *P*_*i*_ with $P_{i}^{*}$ and Replace $f_{P_{i}}$ with $f_{P_{i}^{*}}$.
**Step 6**. If *i* reaches *N*, finish the local search. Otherwise, *i = i*+1 and turn Step 2.

#### GRA-IGSA integration algorithm

When constructing the fitness function, the usual method is to minimize the average simulating error. This method is easy to fall into local optimum and leads to higher simulating accuracy and lower forecasting accuracy. In the proposed VWWBO-BVO-based GM (1,1), the weakened data sequence after pre-processing is used for forecasting. If minimizing the average simulating error of weakened data sequence is taken as the optimization target, the forecasting result will be highly fitted to the weakening data sequence and deviate from the historical annual power load forecasting problem. To avoid these problems, we build the fitness function with the optimization target of maximizing the grey relativity between simulating value and actual value based on GRA.

The original power load sequence is *x*^(0)^ = (*x*^(0)^(1),*x*^(0)^(2),…,*x*^(0)^(*q*)) and the simulating power load sequence based on *x* is ${\hat{y}}^{\left(0\right)}=({\hat{y}}^{\left(0\right)}(1),{\hat{y}}^{\left(0\right)}(2),\ldots ,{\hat{y}}^{\left(0\right)}(q))$. The grey relativity coefficient between *x*^(0)^(*k*) and ${\hat{y}}^{\left(0\right)}(k)$ is as follows:

$$\gamma (x^{\left(0\right)}(k),{\hat{y}}^{\left(0\right)}(k))=\frac{\underset{j=1,2,\ldots ,q}{\text{min}}\left|x^{\left(0\right)}(j)-{\hat{y}}^{\left(0\right)}(j)\right|+\rho \underset{j=1,2,\ldots ,q}{\text{max}}\left|x^{\left(0\right)}(j)-{\hat{y}}^{\left(0\right)}(j)\right|}{\left|x^{\left(0\right)}(k)-{\hat{y}}^{\left(0\right)}(k)\right|+\rho \underset{j=1,2,\ldots ,q}{\text{max}}\left|x^{\left(0\right)}(j)-{\hat{y}}^{\left(0\right)}(j)\right|}$$ (23)

where 0 < *ρ* < 1 is the distinguishing coefficient. Generally, *ρ* = 0.5.

Further, the grey relativity between *x* and ${\hat{y}}^{\left(0\right)}$ is as follows:

$$\gamma (x^{\left(0\right)},{\hat{y}}^{\left(0\right)})=\frac{\sum_{k=1}^{q}\gamma (x^{\left(0\right)}(k),{\hat{y}}^{\left(0\right)}(k))}{q}$$ (24)

Grey relativity reflects the degree of similarity of sequence curves. A bigger grey relativity between simulating power load sequence and original power load sequence shows that simulating power load sequence can better maintain the intrinsic variation of original power load sequence better. The comprehensive simulating and forecasting effect of the proposed model is better. Aiming to maximize the grey relativity between simulating power load sequence and original power load sequence, the fitness function is defined as follows:

$$f[{\left(\lambda ,\eta \right)}_{i}]=\text{max}\left\{\frac{\sum_{k=1}^{q}\gamma (x^{\left(0\right)}(k),{\hat{y}}^{\left(0\right)}(k))}{q}\right\}$$ (25)

Therefore, a GRA-IGSA integration algorithm is proposed. Combining the global optimization ability of GSA and the space expansion ability of local search operator, IGSA algorithm based on local search is formed. Its pseudo codes are as follows:

Begin

*T* = 0;

Initialize the *L*-dimensional population *N*;

 While *T*<*I*

  Calculate the fitness function value of each object;

  Calculate the mass of each object by Eqs (20) and (21);

  Calculate the acceleration of each object by Eq (18);

  Update the velocity of each object by Eq (19);

  Update the mass of each object by Eq (19);

  Do the local search;

  *T*+1;

  End while

End

## Experimental results

### Example analysis

To evaluate the effectiveness of the proposed forecasting model in annual power load forecasting, load data from Literature [28,29] are used for validation. Traditional GM (1,1) model (model 1), GM (1,1) model optimized by buffer operator and time response function (model 2) [20] and the proposed forecasting model (proposed model) are compared and analyzed.

As shown in Table 1, the power consumption data of an area of China from 1998 to 2005 are taken as historical annual power load data for the load forecasting from 2006 to 2008. From Table 1, we can see that the annual power load data have the trend of increasing year by year and fluctuate with economic, climatic and other factors.

**Table 1:** The power consumption data of an area of China from 1998 to 2008 (10^8^KWh).

Year	1998	1999	2000	2001	2002	2003	2004	2005	2006	2007	2008
Power consumption	1.4609	1.5438	1.8239	2.2420	2.2387	2.5059	3.0179	3.4333	3.6265	3.8602	4.1529

The above three models are applied for annual power load forecasting, and percentage error (PE), mean absolute percentage error (MAPE) and grey relativity between simulating value and actual value are used as the indicators to evaluate the above three models. The results are as follows.

**Fig 3:**
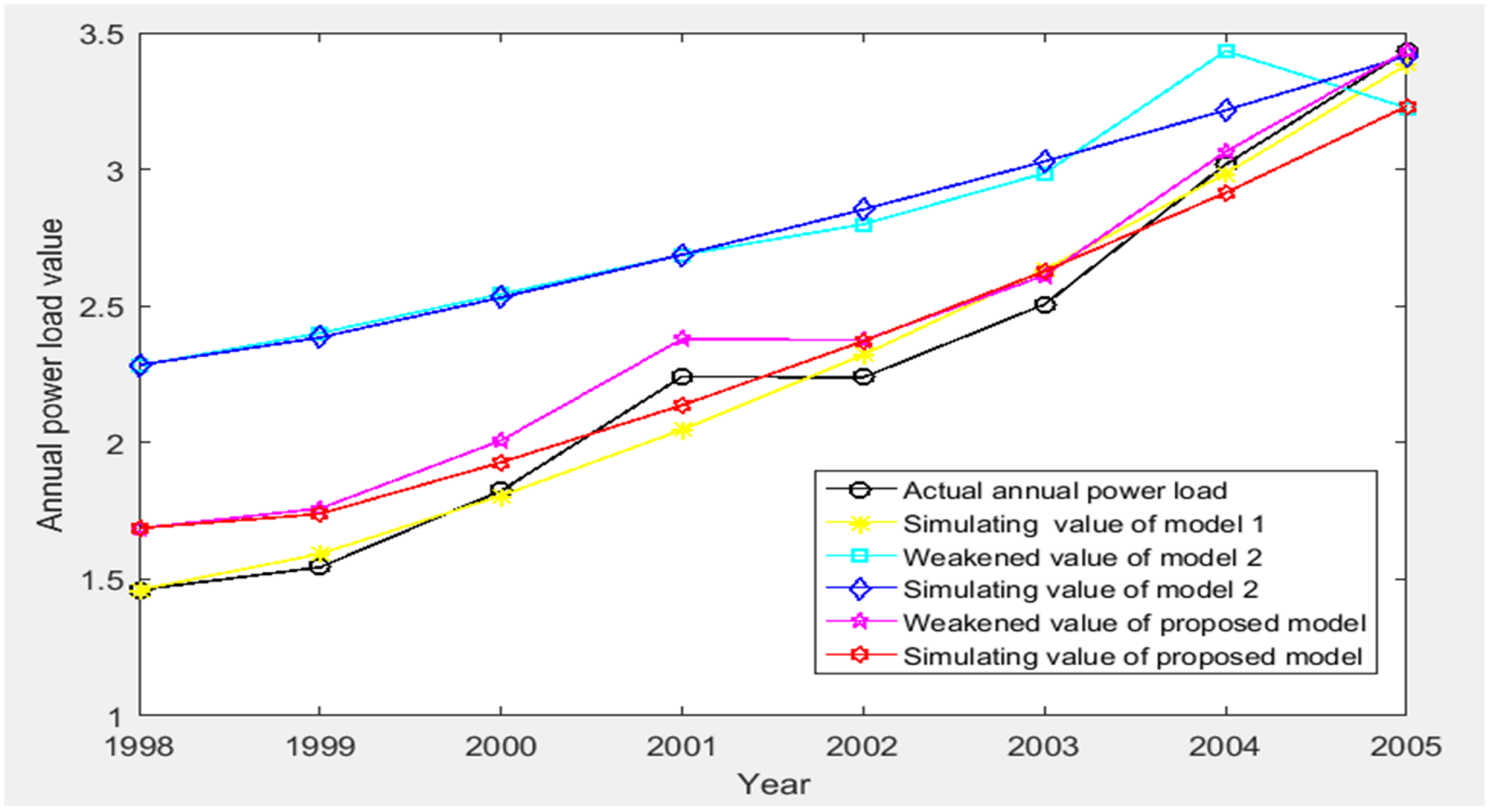
The comparison of the simulating results (1998–2005) of three models.

The simulating results (1998–2005) of three models are shown in Table 2 and Fig 3.

**Table 2:** The simulating results (1998–2005) of three models.

Year		1998	1999	2000	2001	2002	2003	2004	2005
Actual annual power load		1.4609	1.5438	1.8239	2.2420	2.2387	2.5059	3.0179	3.4333
Model 1	Simulating value (10^8^KWh)	1.4609	1.5928	1.8056	2.0472	2.3214	2.6324	2.9845	3.3832
	PE of simulating value relative to actual value (%)	0	3.1740	-1.0033	-8.6887	3.6941	5.0481	-1.1067	-1.4592
Model 2	Weakened value (10^8^KWh)	2.2812	2.4009	2.5437	2.6874	2.7990	2.9859	3.4331	3.2253
	Simulating value (10^8^KWh)	2.2835	2.3847	2.5311	2.6878	2.8531	3.0288	3.2162	3.4146
	PE of simulating value relative to weakened value (%)	0.1008	-0.6747	-0.4953	0.0149	1.9328	1.4368	-6.3179	5.8692
	PE of simulating value relative to actual value (%)	56.3078	54.4695	38.7741	19.8840	27.4445	20.8668	6.5708	-0.5447
Proposed model	Weakened value (10^8^KWh)	1.6859	1.7589	2.0078	2.3791	2.3748	2.6116	3.0647	3.4350
	Simulating value (10^8^KWh)	1.6870	1.7388	1.9279	2.1369	2.3710	2.6273	2.9141	3.2308
	PE of simulating value relative to weakened value (%)	0.0652	-1.1428	-3.9795	-10.1803	-0.1600	0.6012	-4.9140	-5.9447
	PE of simulating value relative to actual value (%)	15.4768	12.6312	5.7021	-4.6878	5.9097	4.8446	-3.4395	-5.8981

**Fig 4:**
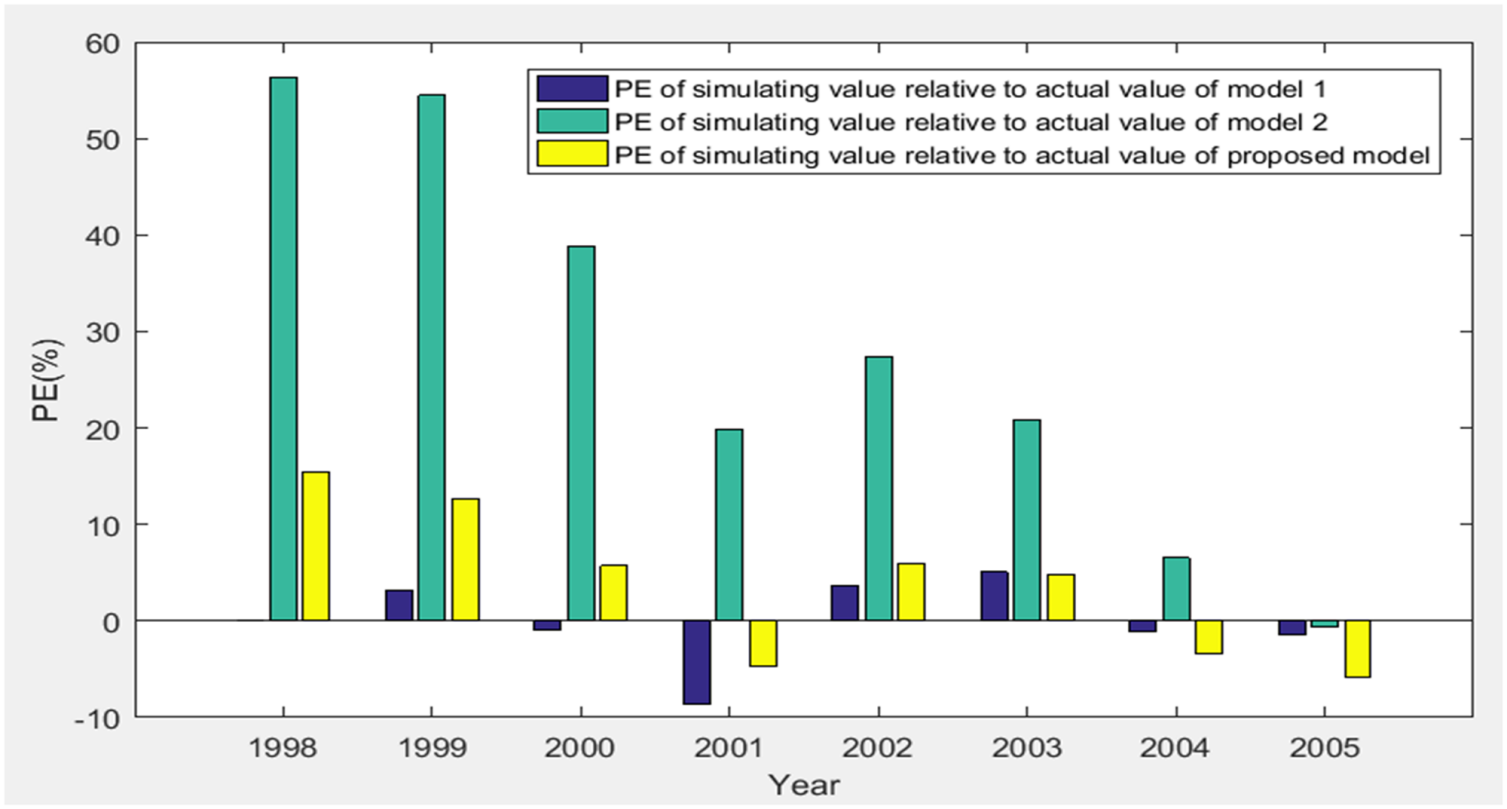
PE of simulating value relative to actual value of three models (1998–2005).

**Fig 5:**
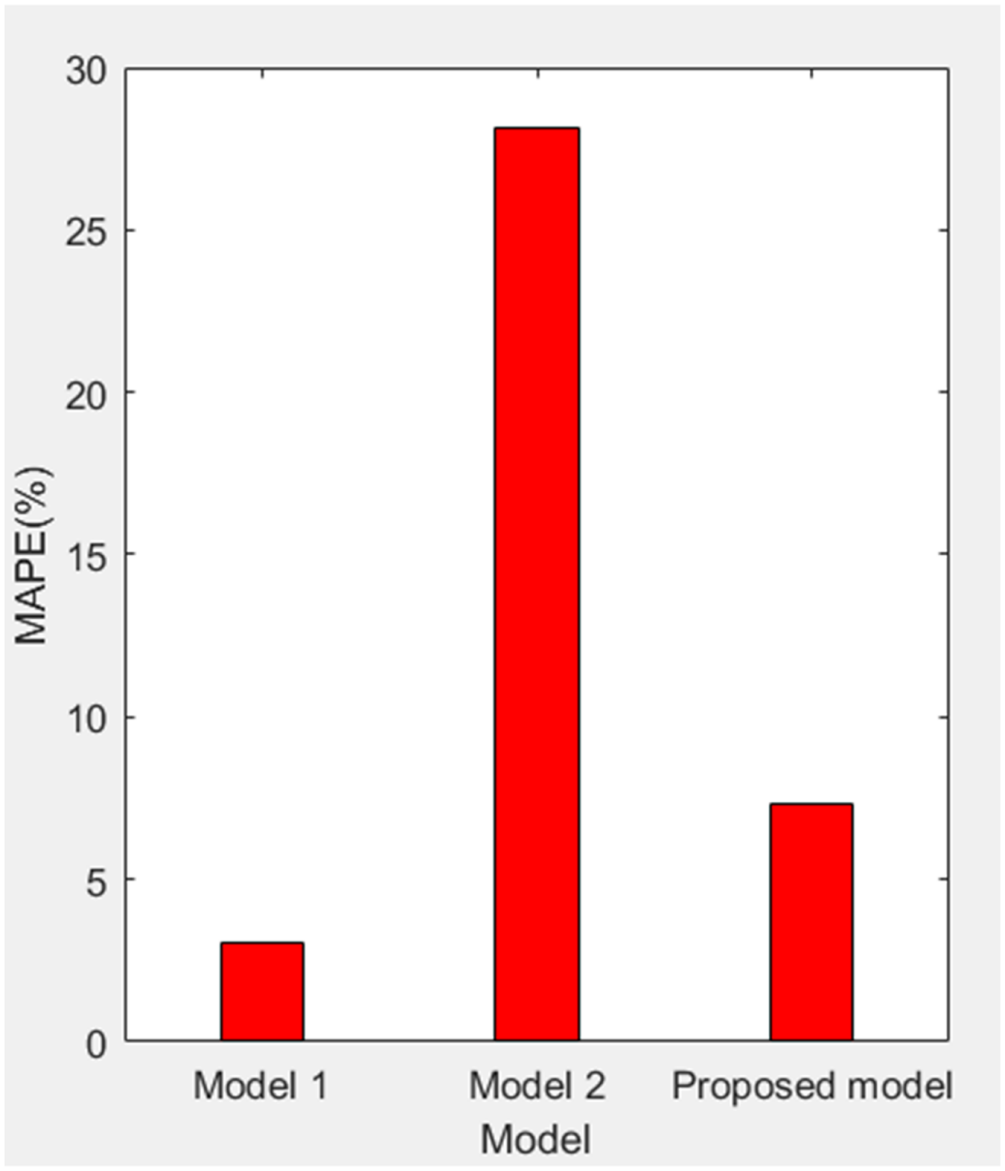
MAPE of simulating value relative to actual value of three models (1998–2005).

**Fig 6:**
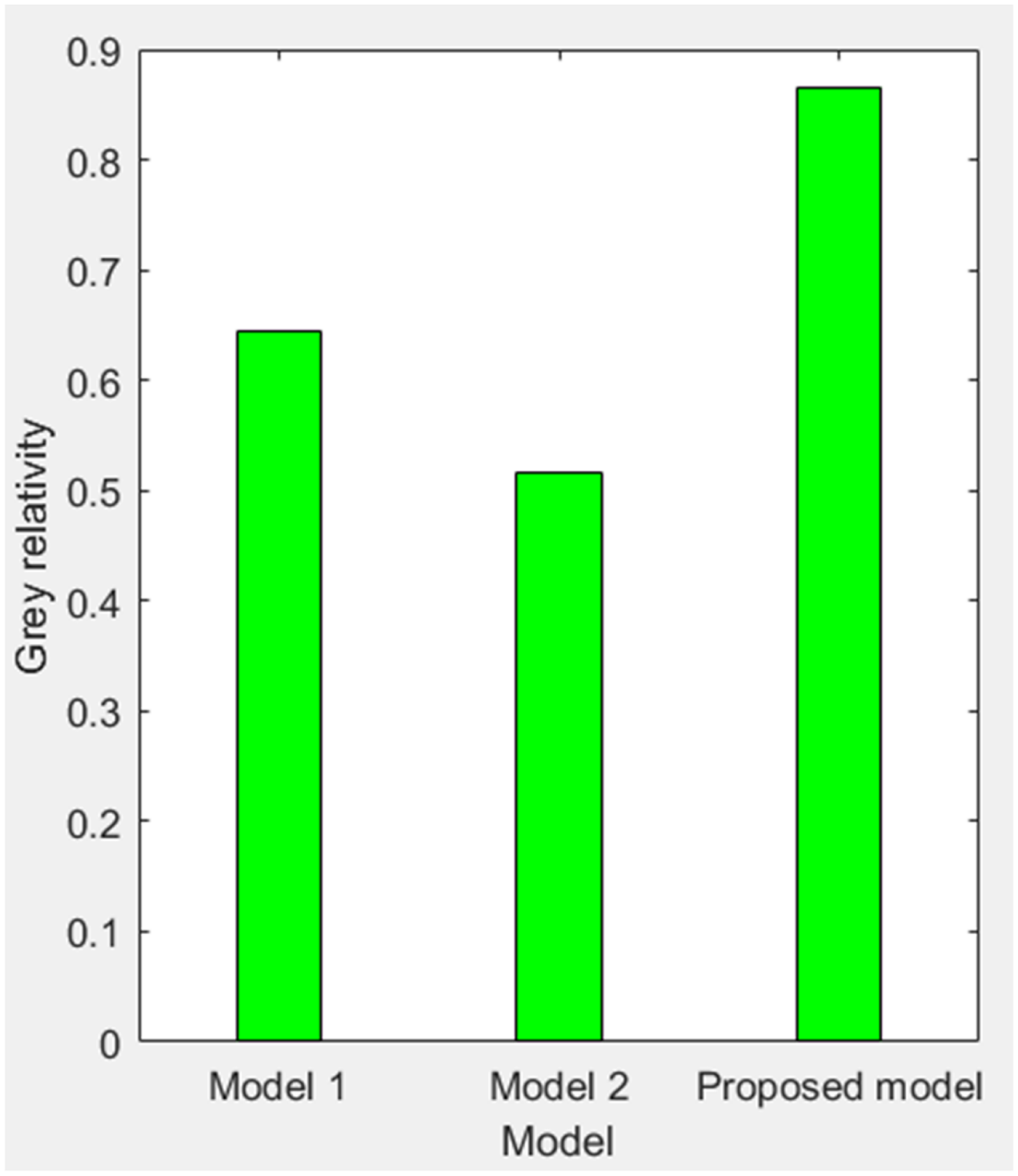
Grey relativity of simulating value relative to actual value of three models (1998–2005).

According to Table 2, MAPE of simulating value relative to actual value of three models are obtained as 3.0218%, 28.1078% and 7.3237%, and the grey relativity of simulating value relative to actual value of three models are obtained as 0.6438, 0.5159 and 0.8662. Therefore, we get the comparisons of PE, MAPE and grey relativity of simulating value relative to actual value of three models in the stage of 1998–2005 as shown in Figs 4–6, respectively.

**Fig 7:**
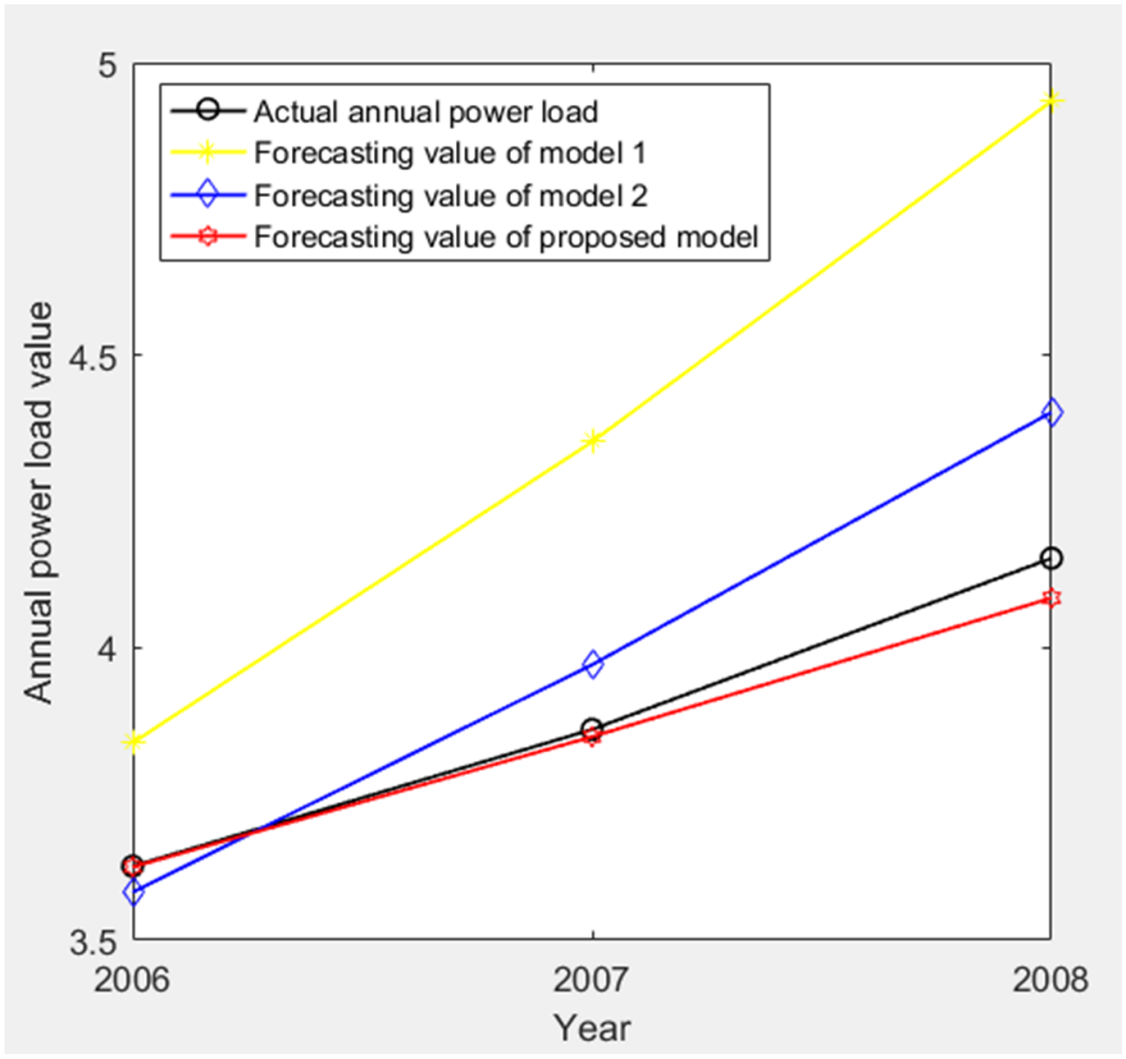
The comparison of the forecasting results (2006–2008) of three models.

The forecasting results (2006–2008) of three models are shown in Table 3 and Fig 7.

**Table 3:** The forecasting results (2006–2008) of three models.

Year		2006	2007	2008
Actual annual power load (10^8^KWh)		3.6265	3.8602	4.1529
Model 1	Forecasting value (10^8^KWh)	3.8373	4.3520	4.9342
	PE of simulating value relative to actual value (%)	5.8128	12.7403	18.8134
Model 2	Forecasting value (10^8^KWh)	3.5822	3.9709	4.4025
	PE of simulating value relative to actual value (%)	-1.2216	2.8677	6.0103
Proposed model	Forecasting value (10^8^KWh)	3.6251	3.8478	4.0849
	PE of simulating value relative to actual value (%)	-0.0386	-0.3212	-1.6374

**Fig 8:**
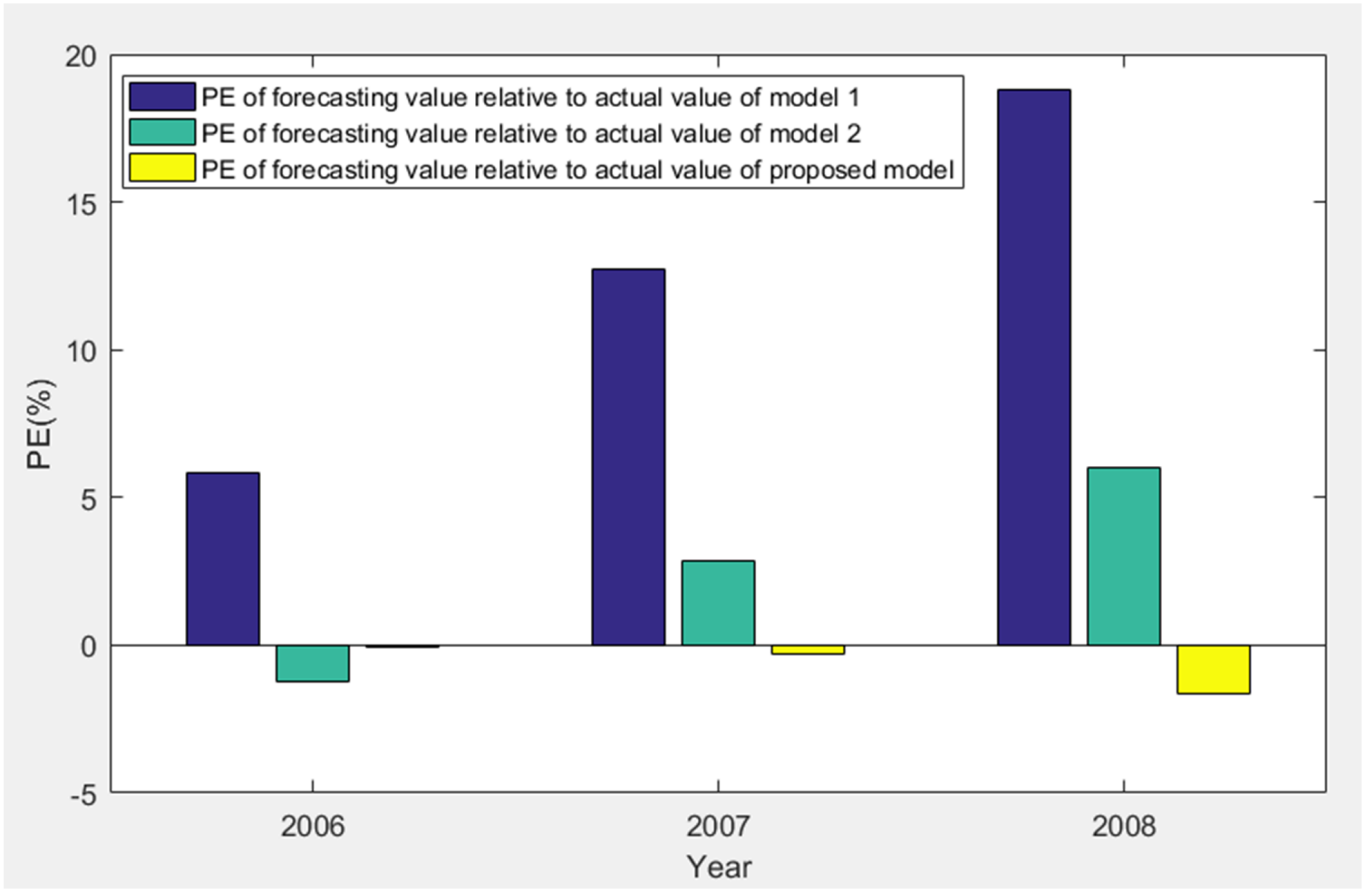
PE of forecasting value relative to actual value of three models (2006–2008).

**Fig 9:**
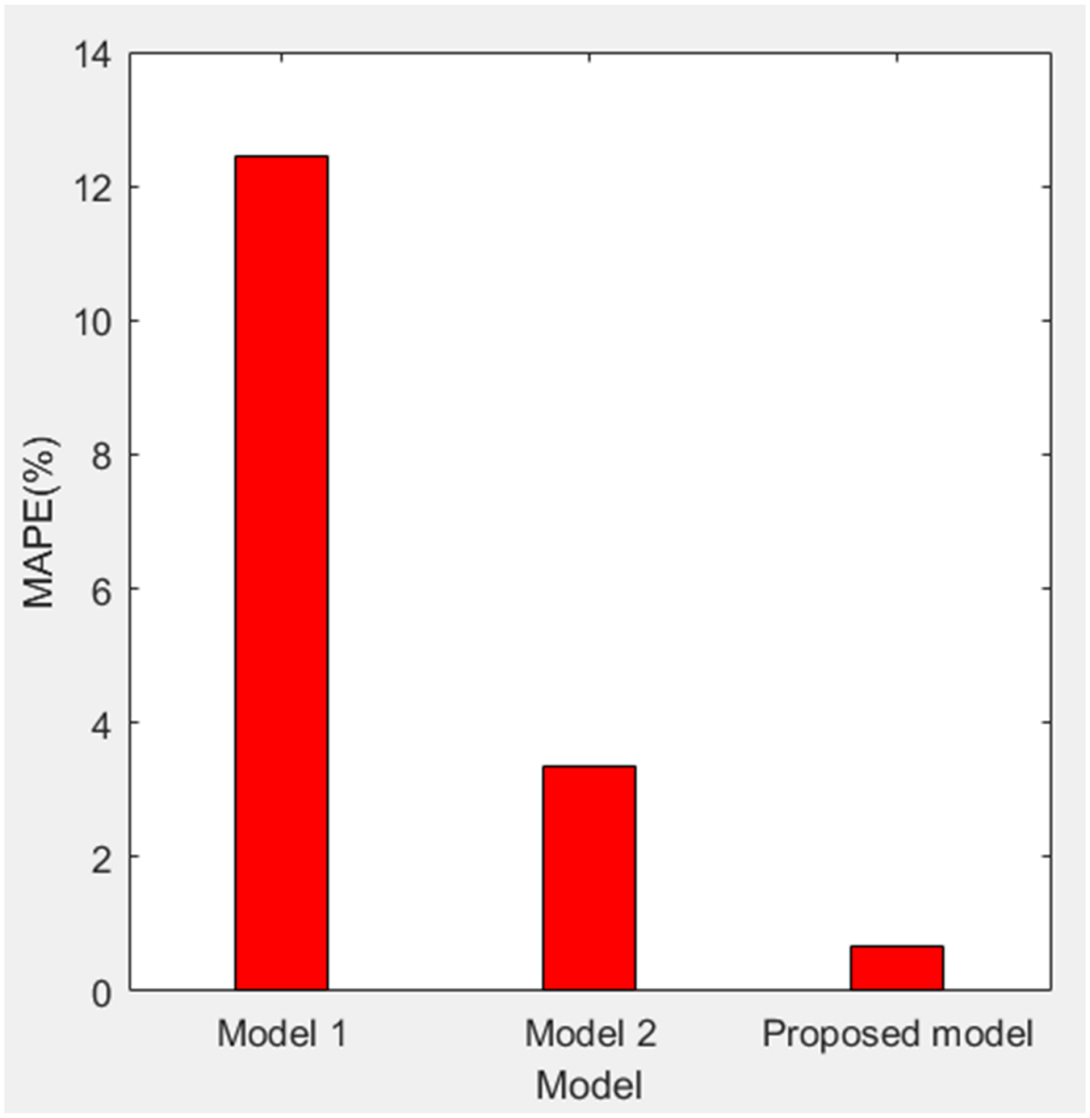
MAPE of forecasting value relative to actual value of three models (2006–2008).

According to Table 3, MAPE of forecasting value relative to actual value of three models are obtained as 12.4555%, 3.3665% and 0.6657%. Therefore, we get the comparisons of PE and MAPE of forecasting value relative to actual value of three models in the stage of 2006–2008 as shown in Figs 8 and 9, respectively.

Based on Figs 3–9, model 1, model 2 and proposed model are compared and analyzed as follows:

- For model 1, MAPE of simulating value relative to actual value of three models (1998–2005) is 3.0218% (in Fig 5), so the simulating effect is good. However, it is a failure to control and weaken the fluctuations in historical annual power load sequence (actual value in Table 1). The forecasting effect is poor and MAPE of forecasting value relative to actual value (2006–2008) reaches 12.4555% (in Fig 9).
- For model 2, mean weakening buffer operator is used to pre-process the historical annual power load sequence and GM (1,1) model optimized by time response function is built. High accuracy simulating to weakened value is realized as shown in Fig 3, but MAPE of simulating value relative to actual value (1998–2005) reaches 28.1078% (in Fig 5) and the grey relativity is only 0.5159 (in Fig 6). This illustrates that the buffering effect of mean weakening buffer operator is too strong. The main trend and fluctuation law of weakened value sequence and forecasting value sequence will be completely different from historical annual power load sequence. It can be considered that the high accuracy simulating and forecasting object of model 2 is just the weakened value sequence after pre-processing. In fact, it deviates from the intrinsic annual power load forecasting problem.
- For proposed model, IGSA is used to maximize the grey relativity between simulating value sequence and actual value sequence. The optimal value of variable-weight weakening buffer coefficient *λ* and background-value weight generating coefficient *η* are found to do the pre-processing of the historical annual power load data, and then the VWWBO-BVO-based GM (1,1) is built for annual power load forecasting. The basic parameters of IGSA are set as: the initial value of gravitational constant *G*_0_ = 10, the constant *l* = 1.0, the number of objects *N* = 40, the number of iterations *T* = 300. The optimal value are found as *λ* = 0.1136 and *η* = 0.7659 by GRA-IGSA integration algorithm. By proposed model, MAPE of simulating value relative to actual value (1998–2005) is 7.3237% (in Fig 5) and the grey relativity of simulating value relative to actual value reaches 0.8662 (in Fig 6). MAPE of forecasting value relative to actual value (2006–2008) is 0.6657%, which is the lowest in three models (in Fig 9).

Compared with model 1, proposed model uses buffer operator for historical annual power load sequence pre-processing, weakens the random fluctuation of historical annual power load sequence, and strengthens the main development trend. Although the simulating accuracy is slightly worse, a high forecasting accuracy is achieved. Compared with model 2, proposed model aims to maximize the grey relativity of simulating value relative to actual value and uses GRA-IGSA integration algorithm to optimize the selection of weakening buffer operator. The problem of nonadjustable buffering effect has been solved. While using the buffer operator to pre-process the historical annual power load sequence to improve the forecasting accuracy, the inherent trend and the fluctuation law of historical annual power load sequence are maintained to the maximum extent. The simulating accuracy is improved by an order of magnitude (in Figs 4 and 5). Therefore, the proposed model is more consistent with the historical annual power load forecasting problem and has more practical significance.

### Practical application

**Fig 10:**
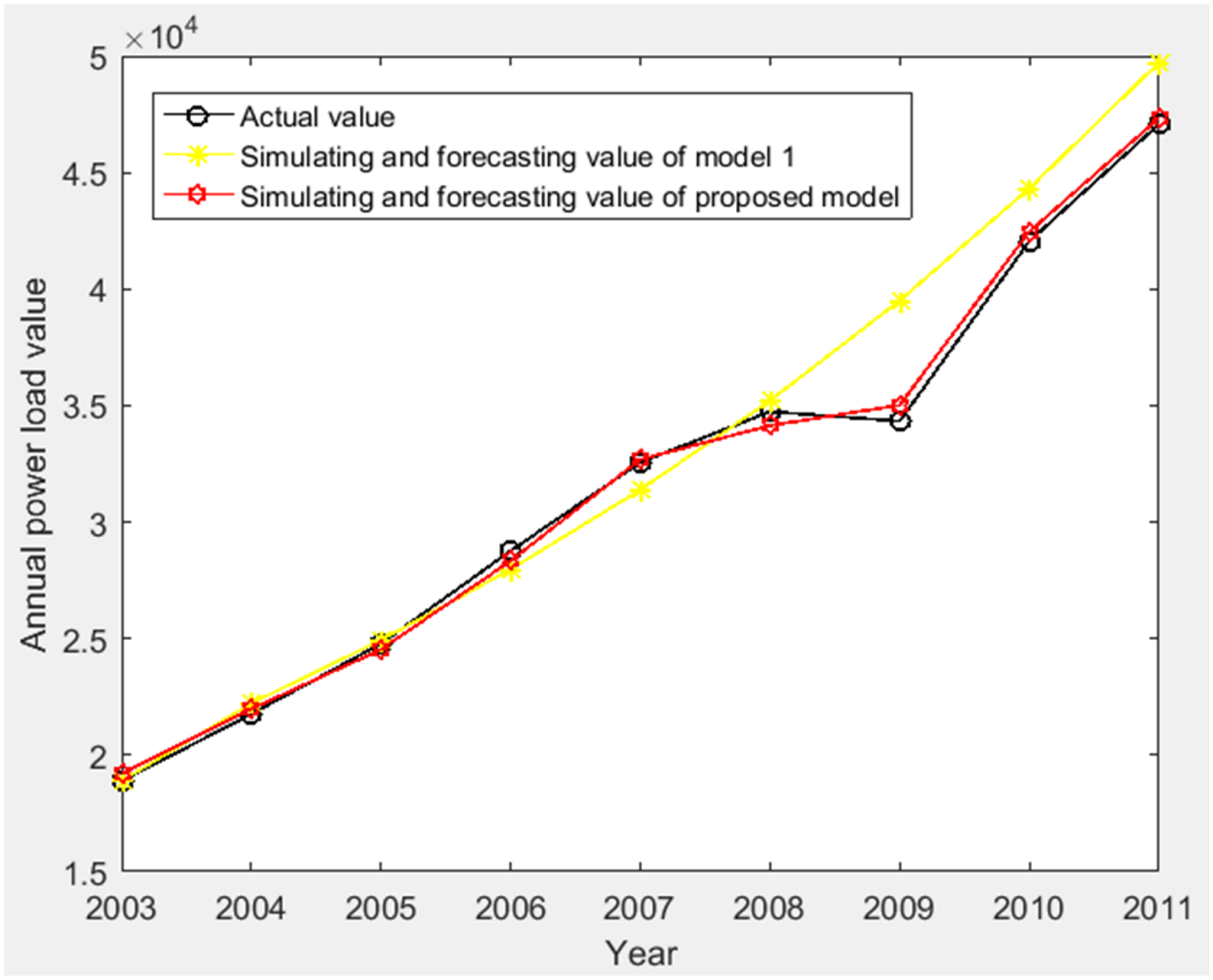
The comparison of the simulating and forecasting results for nationwide annual power load of model 1 and proposed model (2003–2011).

Using the nationwide power consumption of China (2003–2008) [29,30] as the historical annual power load data, we forecast the nationwide power consumption in 2009–2011. In 2003–2007, China shook off the impact of the 1998 financial crisis. The economy was booming and the nationwide power consumption increased by an average of 13.16%. In 2008, the emergence of major natural disasters and the 2008 financial crisis had a great impact on power consumption. Although power consumption continued to grow, the growth rate has dropped considerably. Overall, annual power consumption data in 2003–2008 years shows the main trend of sustained growth. Due to economic factors, natural disasters and other effects, it also shows some fluctuations. Therefore, using the proposed model for annual power load forecasting is suitable. The basic parameters of IGSA are set as same as Section Example analysis. The simulating results and forecasting of model 1 and proposed model are shown in Table 4 and Fig 10.

**Table 4:** The nationwide power consumption of China (2003–2011) and the simulating and forecasting results of model 1 and proposed model.

Year		2003	2004	2005	2006	2007	2008	2009	2010	2011
Actual annual power consumption (10^8^KWh)		18891	21761	24781	28768	32565	34739	34333	42055	47081
Model 1	Simulating value (10^8^KWh)	18891	22224	24934	27974	31385	35212	/	/	/
	Forecasting value (10^8^KWh)	/	/	/	/	/	/	39506	44323	49727
	PE of simulating value relative to actual value (%)	0	2.1277	0.6174	-2.7600	-3.6235	1.3616	/	/	/
	PE of forecasting value relative to actual value (%)	/	/	/	/	/	/	15.0671	5.3929	5.6201
Proposed model	Simulating value (10^8^KWh)	19201	21981	24533	28337	32688	34145	/	/	/
	Forecasting value (10^8^KWh)	/	/	/	/	/	/	35016	42467	47333
	PE of simulating value relative to actual value (%)	1.6410	1.0110	-1.0008	-1.4982	0.3777	-1.7099	/	/	/
	PE of forecasting value relative to actual value (%)	/	/	/	/	/	/	1.9893	0.9797	0.5352

According to Table 3, MAPE of forecasting value relative to actual value of three models are obtained as 12.4555%, 3.3665% and 0.6657%. Therefore, we get the comparisons of PE and MAPE of forecasting value relative to actual value of three models in the stage of 2006–2008 as shown in Figs 8 and 9, respectively.

According to Table 4, MAPE of simulating value relative to actual value (2003–2008) of model 1 and proposed model are 1.7484% and 1.2064%, and MAPE of forecasting value relative to actual value (2009–2011) of model 1 and proposed model are 8.6934% and 1.1681%. Additionally, the grey relativity (2003–2008) of model 1 and proposed model are 0.6168 and 0.8366. By the proposed model, the intrinsic variation law of the actual annual power load sequence is better grasped, the forecasting accuracy is greatly improved, and the simulating and forecasting results are stable. Therefore, the proposed model can meet the requirements of annual power load forecasting.

## Conclusions

The main findings of this study are summarized as follows.

1. The proposed model introduces variable-weight weakening buffer operator to dynamically pre-process the original power load data and realizes the action intensity adjustment of buffer operator by variable weight buffer coefficient. It effectively solves the problem of nonadjustable action intensity of traditional buffer operator, enhances the adaptability and improves the forecasting precision.
2. Based on grey relational analysis and improved gravitational search algorithm, the proposed model aims to maximize the grey relativity between simulation value sequence and actual value sequence for parameter optimization.
3. The intrinsic variation law of the historical annual power load data is maintained to the maximum extent. It realizes the organic unification of data pre-processing and historical annual power load forecasting, so the simulation and forecast effect are more stable and the forecast accuracy is higher.

Except for the proposed model, a variety of variable-weight buffer grey model can be constructed based on the variable-weight buffer operators with different structures. The continuous improvement of forecasting accuracy and the adaptability in annual power load forecasting of different curve types will be the next research contents.
